# Atypical presentation of immunoglobulin G4-related disease as subglottic stenosis: a case-based review

**DOI:** 10.1007/s00296-021-04816-4

**Published:** 2021-04-08

**Authors:** Belén Atienza-Mateo, Teresa Díaz de Terán-López, Javier Gómez-Román, Laura Sánchez, Roberto Mons-Lera, Antonio Rubio-Suárez, José Manuel Cifrián, Miguel Á. González-Gay

**Affiliations:** 1 Division of Rheumatology, Hospital Universitario Marqués de Valdecilla, Avda. Valdecilla s/n, 39008 Santander, Spain; 2 Epidemiology, Genetics and Atherosclerosis Research Group On Systemic Inflammatory Diseases, IDIVAL, Santander, Spain; 3 Division of Pulmonology, Hospital Universitario Marqués de Valdecilla, Santander, Spain; 4 Division of Pathology, Hospital Universitario Marqués de Valdecilla, Santander, Spain; 5 University of Cantabria - IDIVAL, Santander, Spain; 6 Division of Thoracic Surgery, Hospital Universitario Marqués de Valdecilla, Santander, Spain; 7 Division of Otorhinolaryngology, Hospital Universitario Marqués de Valdecilla, Santander, Spain; 8 Cardiovascular Pathophysiology and Genomics Research Unit, School of Physiology, Faculty of Health Sciences, University of the Witwatersrand, Johannesburg, South Africa

**Keywords:** Immunoglobulin G4-related disease, Subglottic stenosis, Rituximab, Review

## Abstract

Immunoglobulin G4-related disease (IgG4-RD) is a recently recognized fibro-inflammatory pathology that has been reported to affect principally the retroperitoneum, hepatobiliary system, salivary glands, orbital structures or lymph nodes. However, IgG4-RD with laryngeal involvement is a very rare entity. Our aims were to describe a case of subglottic stenosis as first and only manifestation of IgG4-RD and review the literature. A patient with IgG4-RD affecting the larynx that presented as subglottic stenosis is described. A MEDLINE database search of IgG4-RD cases with laryngopharyngeal manifestations was also conducted. A 30-year-old Caucasian woman was referred to a tertiary care hospital for dyspnea on exertion, which had been increasing for the last 4 months. Medical and surgical procedures revealed a subglottic stenosis, with a histological finding of IgG4 positive plasma cell infiltration. There was no evidence of other organ involvement. She was successfully treated with oral glucocorticoids and rituximab infusions. Glucocorticoids were rapidly tapered and the rituximab regimen was optimized, with no evidence of relapses. In the literature review, we found a total of 12 reported cases with laryngopharyngeal involvement, two of them with subglottic stenosis. IgG4-RD of the larynx is rare but should be considered after excluding more common disorders.

## Introduction

Immunoglobulin G4-related disease (IgG4-RD) is an idiopathic fibro-inflammatory disorder characterized by the presence of swelling or masses in single or multiple organs, with specific clinical, serological and histopathological features [1, 2]. The pathological hallmark of this disease is a dense lymphoplasmacytic infiltrate with IgG4 positive plasma cells (at least 10 per high power field with ratio of IgG4 + /IgG + cells > 40%), storiform fibrosis and obliterative phlebitis [3]. Laboratory results include elevated serum IgG4 levels (greater than 135 mg/dl) and non-specific findings like increased total IgG and IgE, hypocomplementemia, peripheral eosinophilia, elevation of acute phase reactants, anti-nuclear antibody positivity or raised rheumatoid factor levels [4]. However, serum IgG4 concentration can be normal in up to 30–50% of the patients [5, 6]. IgG4-RD often involves the pancreas, hepatobiliary system, orbital structures, salivary glands, lymph nodes or retroperitoneum. Besides, any organ can be affected [7] and atypical presentations of this condition are not uncommon [8].

In this report, we describe the case of a young woman diagnosed with IgG4-RD, who had subglottic stenosis as the only manifestation. A literature review of patients with IgG4-RD with laryngopharyngeal involvement was also conducted.

## Case presentation

A 30-year-old Caucasian woman was referred to a tertiary care hospital (XXX) in June 2016 for dyspnea on exertion, which had been increasing for the last 4 months. She had been diagnosed of laryngitis and treated with a short course of oral glucocorticoids at her primary care center. She was not a smoker and had no relevant family or medical history or known allergies. She had never been intubated, but she underwent adenoidectomy in childhood.

**Fig. 1:**
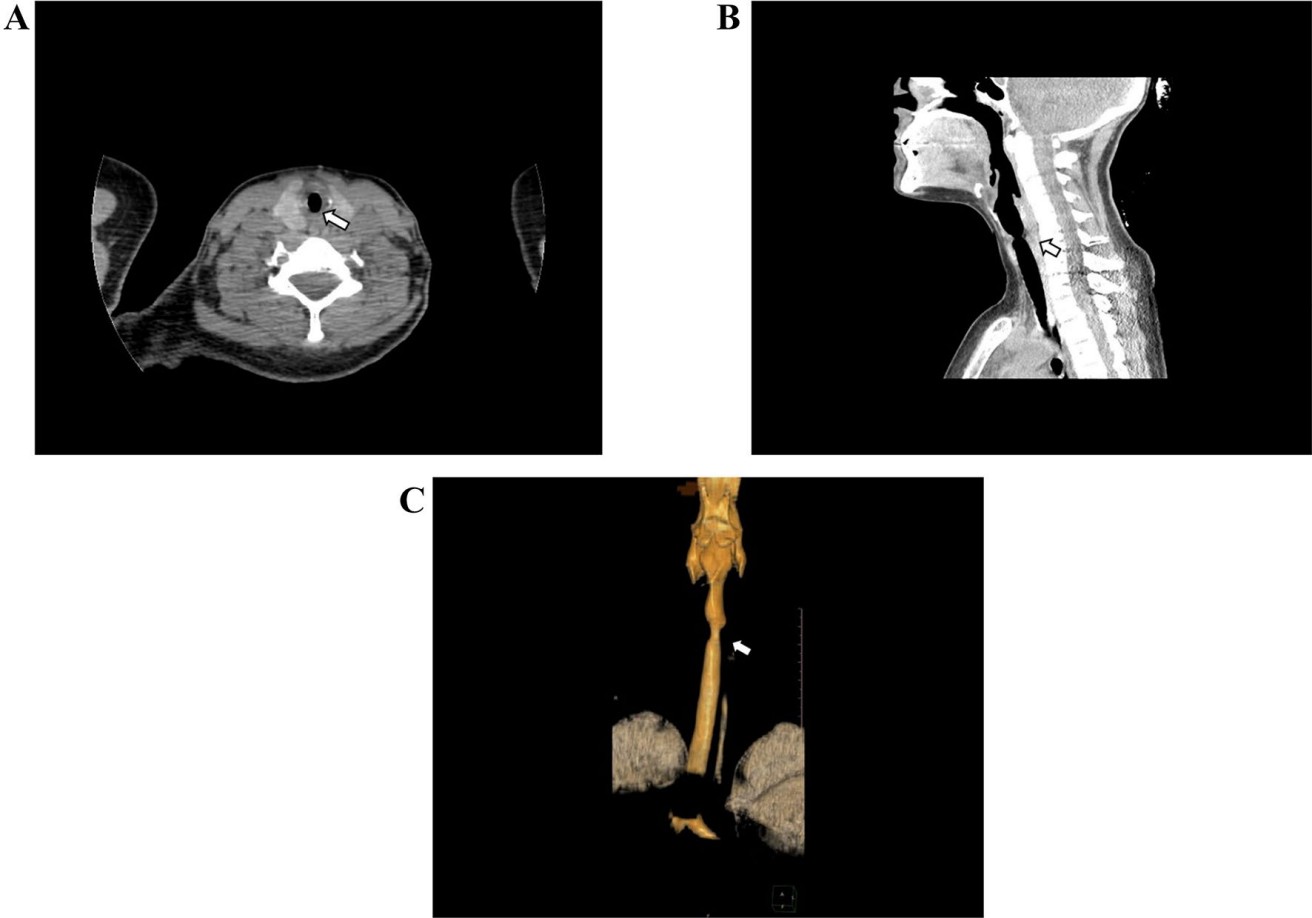
Cervical computed tomography images showing a thickening at the level of the first tracheal ring (arrows), with no apparent origin. **a** Axial plane. **b** Sagittal plane. **c** 3D reconstruction

**Fig. 2:**
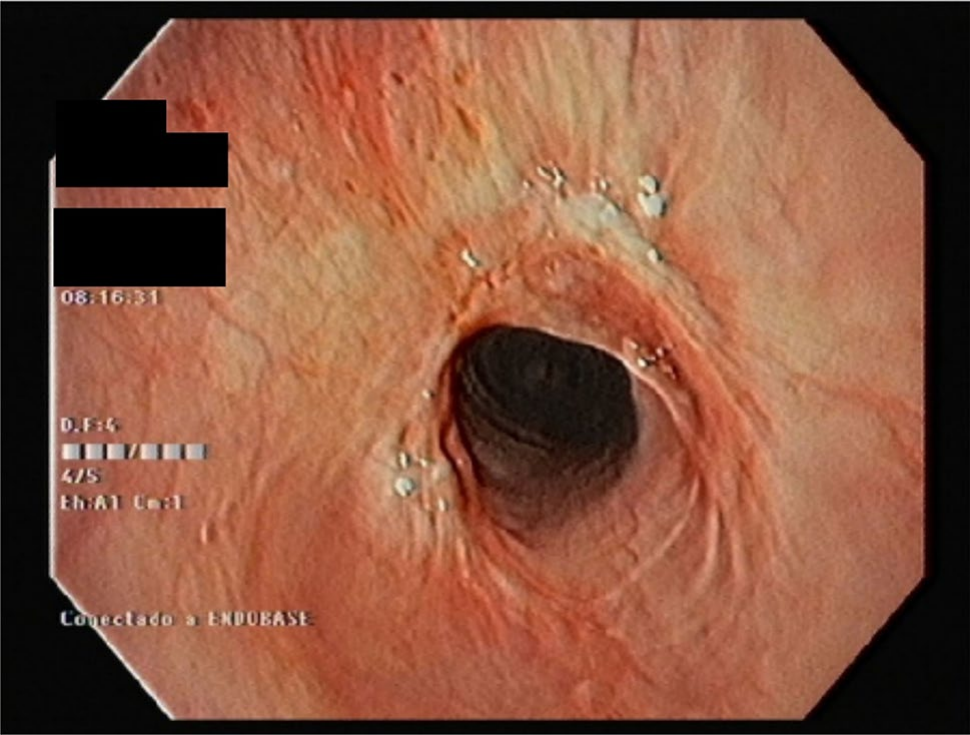
Rigid bronchoscopy image of larynx after dilation of the lumen

**Fig. 3:**
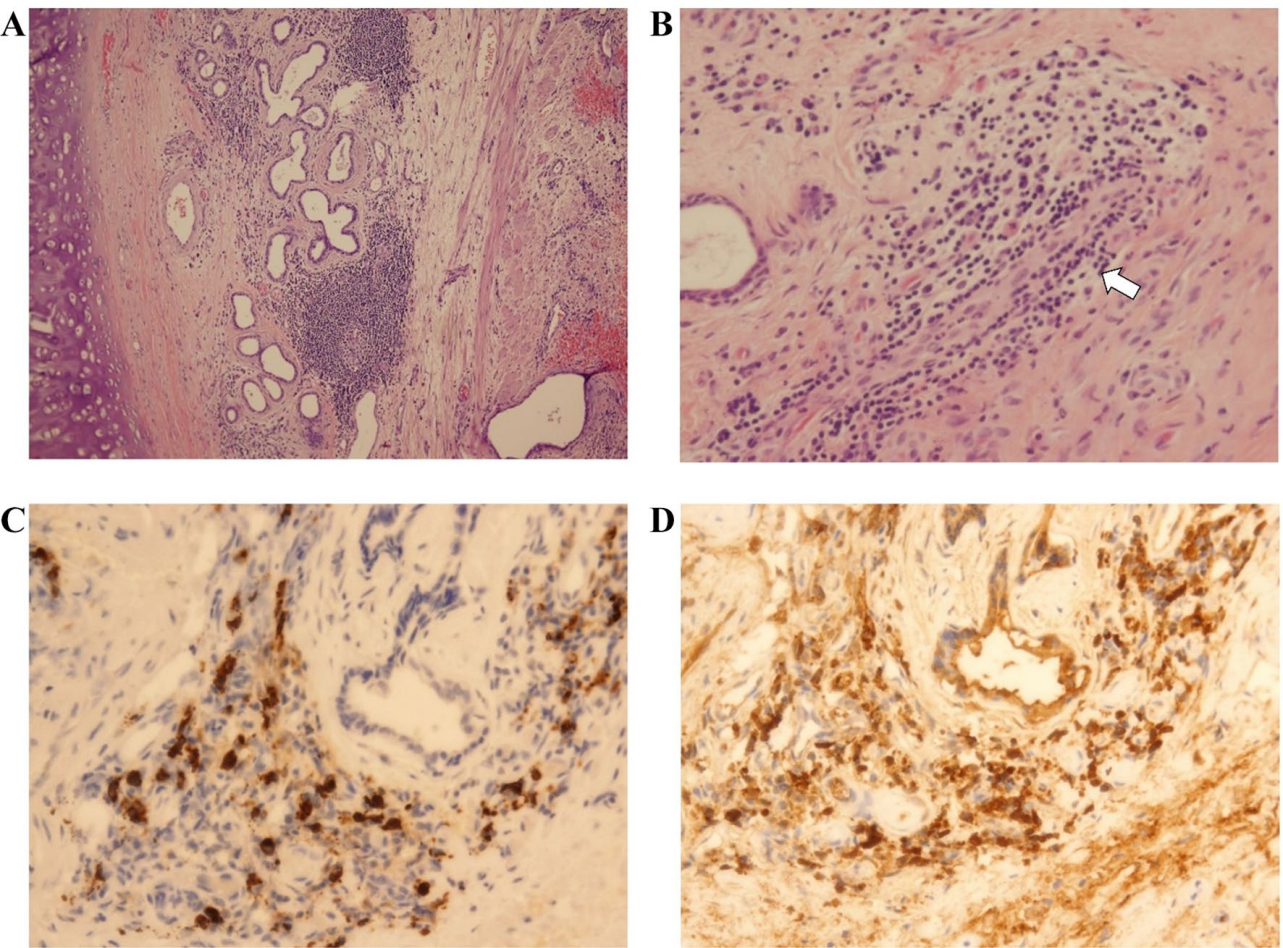
Histopathological examination of the tracheal biopsy. **a** Note a submucosal inflammatory lymphoid nodular tissue destroying seromucous glands (Hematoxylin and Eosin [H&E] Stain, original magnification × 25). **b** The lymphoid infiltrate affects small arteries in an endarteritis obliterans pattern (arrow) (H&E Stain, original magnification × 100). **c **and **d** Immunohistochemistry performed with monoclonal antibodies against IgG4 (**c**) and IgG (**d**) demonstrates nodular aggregates of IgG4 + plasma cells in more than 25% of total IgG + plasma cells (Original magnification × 100)

**Fig. 4:**
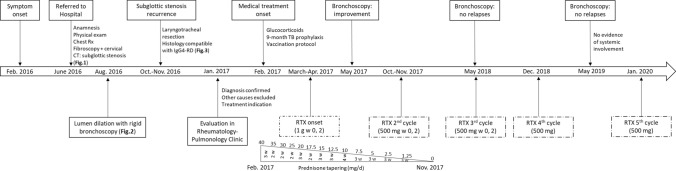
Timeline of diagnostic and therapeutic events of our patient. *CT* computed tomography, *IgG4-RD* Immunoglobulin G4-related disease, *TB* tuberculosis, *RTX* rituximab, *RX* simple radiography, *w* week(s)

Her physical examination and chest radiograph were normal. However, when a laryngeal fibroscopy was performed, a membranous subglottic abnormality occluding 50% of the lumen was found. Subsequent computed tomography of the neck showed a decreased caliber of the proximal trachea beginning 5 mm below the cricoid cartilage and extending 6 mm distally between the thyroid lobes (Fig. 1). The patient was then evaluated by Thoracic Surgery Service and a rigid bronchoscopy with dilation to a lumen of 80% was performed (Fig. 2). Two months later, the patient suffered a recurrence of the subglottic stenosis, for which she underwent laryngotracheal resection. The histology of the surgical specimens revealed a chronic fibrotic inflammatory infiltrate with IgG4 hyperproduction in the cricoid ring as well as the first to fourth tracheal rings. The IgG4 + /IgG + plasma cell ratio was 25%. The histopathologic findings are shown in Fig. 3 (see Fig. 4).

In light of these findings, the patient was evaluated in a combined Rheumatology-Pulmonology clinic, where it was confirmed that no additional organs were involved (no thyroid disorders, pancreatitis, abdominal problems, sicca syndrome, polymyalgia or other autoimmune features).

Laboratory tests including full blood cell count, biochemistry profile and serologies (microbiological, complement, rheumatoid factor, antinuclear antibodies, anti-neutrophil cytoplasmic, anti-phospholipid and anti-thyroid antibodies) were within the normal range or negative, as appropriate for each case. Serum IgG4 was also normal at baseline (48.00 mg/dL) and during follow-up (maximum of 55.61 md/dL). Apart from glucocorticoids (initial dose 40 mg/day), intravenous rituximab (RTX) was started with a regimen infusion of 1000 mg on days 0 and 14 (previously administered intravenous methylprednisolone and dexchlorpheniramine). Before RTX initiation, the patient underwent a vaccination program and was treated with isoniazid/pyridoxine (300 mg/50 mg per day) the previous month and for a total of 9 months due to Quantiferon test positivity.

Posterior medical assessments and fibroscopic examinations showed a remarkable improvement of the patient, with no further signs of subglottic stenosis or laryngeal inflammation and no systemic manifestations. Moreover, a 18F-FDG PET/CT (fluorine-18-fluorodeoxyglucose positron emission tomography/computed tomography) scan confirmed the absence of vascular or other organ involvement. Prednisone was gradually tapered up to complete discontinuation after 9 months since the onset of this therapy. Also, RTX therapy was optimized reducing half the dose to 500 mg on days 0 and 14 and subsequently to 500 mg every 6 months and later 500 mg annually. At present, 4 years after the diagnosis, she is on annual cycles of RTX with no relapses.

## Search strategy and literature overview

A comprehensive search of biomedical literature until December 2020 about cases of IgG4-RD patients with laryngotracheal involvement was performed. The research sources consulted were from MEDLINE, life science journals and online books published primarily on PubMed. The search was conducted using the following terms: “IgG4-related disease” and “trachea” or “larynx” or “pharynx”. The references in each selected study were reviewed to identify other relevant articles. Scopus and Web of Science databases were also searched with no additional related articles found. Two authors reviewed the search results and agreed on the included articles. From each article selected, data were collected on publication year, gender, age, location of the IgG4-RD, treatment received and time of follow-up. Non-English articles were excluded for the review.

## Results

We identified 11 reports describing cases of laryngotracheal involvement due to IgG4-RD [16–26], 2 of them written in a non-English language [22, 25]. Table 1 includes an update of the published cases written in English [16–21, 23, 24, 26]. We found a total of 12 patients with laryngopharyngeal involvement, most of them adult men. The most common sites of IgG4 + plasma cell infiltration were the pharynx [16, 19, 21] and the supraglottic region of the larynx [17, 20, 21, 24]. Treatment strategies included local surgery and immunosuppressive drugs.

**Table 1:** Literature review of published cases of IgG4-RD patients with laryngotracheal involvement

Authors and publication year	N	Gender	Age^a^	IgG4-RD location	Treatment	Follow-up^b^
Masterson et al. 2010 [16]	1	Women	58	Pharynx, gallbladder, lungs, pelvis, omentum, eyes and left temporal bone	Several surgeries + GC + MMF	12
Völker et al. 2010 [17]	1	Male	56	Supraglottis	Laser resection + GC	24
Virk et al. 2012 [18]	1	Woman	22	Subglottis	GC + surgical reconstruction	ND
Shaib et al. 2013 [19]	2	Male Male	56 57	Subglottis and lungs Pharynx and larynx	Laser therapy + GC + AZA Surgical resection + GC	24 ND
Khoo et al. 2014 [20]	1	Male	62	Supraglottis	GC	ND
Reder et al. 2015 [21]	3	Male Male Woman	58 62 50	Supraglottis Supraglottis Supraglottis and pharynx	GC + RTX GC + RTX RTX	24 12 15
Suárez-Díaz et al. 2020 [23]	1	Woman	37	Larynx	GC + AZA	ND
Matsushima et al. 2020 [24]	1	Male	50	Supraglottis, ureters	GC + ureteral stents	ND
Syed et al. 2020 [26]	1	Male	69	Larynx, lagrimal glands, pancreas	GC + RTX	24
Atienza-Mateo et al. present case	1	Woman	30	Subglottis	Surgical resection + GC + RTX	48

*AZA* azathioprine, *GC* glucocorticoids, *IgG4-RD* immunoglobulin G4-related disease, *MMF* mycophenolate mofetil, *N* number of patients, *ND* no data, *RTX* rituximab

^a^Age is expressed in years. ^b^Follow-up stands for minimum follow-up reported after medical treatment initiation and is expressed in months

## Discussion

IgG4-RD is a relatively recently recognized multi-organ disease that manifests with pseudotumoral masses of inflammatory fibrosis in the pancreas, salivary glands, hepatobiliary system or eye orbit, among others [1, 2]. The exact pathogenesis remains unclear; however, components of both autoimmune and allergic dysfunction are thought to be involved [9]. The definitive role of IgG4 molecule is still unknown, whether this subclass of IgG immunoglobulin has a direct implication in the pathophysiology of the disease or is an epiphenomenon in other processes [10]. Nonetheless, the infiltration of positive IgG4 plasma cells in the involved organ is required to make a definite diagnosis of IgG4-RD [3]. The histological results of our patient’s affected tissue shed light on the diagnosis of IgG4-RD since typical features were found in the examination of the samples (Fig. 3).

The presence of high serum concentration of IgG4 immunoglobulins can be useful in reinforcing the diagnosis but is not required to be present or self-sufficient for the diagnosis [11]. It is usually found in patients with multiple organ damage and up to 30–50% of the patients have normal IgG4 serum levels, as in our case [5, 6].

Glucocorticoids are the first line of treatment and they should be administered urgently when vital organs are affected [12]. However, glucocorticoid monotherapy usually fails to control the disease activity and entails several long‐term adverse effects. In this regard, RTX, a monoclonal chimeric antibody against the surface antigen CD20, expressed on pre-B and B lymphocytes, has shown efficacy in the treatment of patients with IgG4-RD, even as induction therapy without concomitant glucocorticoids [13]. Thus, RTX therapy should be considered as an effective and glucocorticoid-sparing agent in patients with IgG4-RD [12]. Other immunosuppressive drugs such as azathioprine, methotrexate or mycophenolate mofetil have also been used [14]. However, the efficacy of these agents has not been evaluated in prospective trials. Our patient was treated with initial doses of 40 mg/day of prednisone plus an infusion of 1000 mg of intravenous RTX on days 0 and 14 (previously administered intravenous methylprednisolone and dexchlorpheniramine). Following this therapy, glucocorticoids were tapered and discontinued in 9 months. The optimal maintenance therapy duration for IgG4-RD has not been clearly defined. In our case, RTX administration was optimized after induction regimen, reducing half the dose to 500 mg and later spacing the administration interval with a successful outcome.

IgG4-RD comprises a group of heterogeneous and complex entities. In this sense, the 2019 American College of Rheumatology/ European League Against Rheumatism classification criteria for IgG4-RD [15] have been developed to compile clinical, serological, radiological and pathological features associated with this disease to properly categorize patients and facilitate high-quality clinical and epidemiological studies. These criteria include 11 organs that are typically involved in IgG4-RD: pancreas, bile ducts, orbits, lacrimal glands, major salivary glands, retroperitoneum, kidneys, lungs, aorta, pachymeninges and thyroid gland. However, they do not include the larynx o the pharynx. There are few recorded cases of this disease presenting with involvement of the laryngopharynx. We made a comprehensive literature review of IgG4-RD patients with laryngopharyngeal manifestations. It is noteworthy that, in addition to our case, only two other patients with subglottic stenosis have been reported [18, 19]. Both were treated with glucocorticoids and underwent surgery. One of them was similar to our case. In this regard, Virk JS et al. reported a 22-year-old woman with a large history of “idiopathic subglottic stenosis”, in whom a diagnosis of IgG4-RD was made 3 years later by means of biopsy after thoracotomy. Two months following glucocorticoid treatment initiation (not information on dose was available), the patient underwent laryngotracheal reconstruction. She remained with normal voice and minimal dyspnea. Our present case was diagnosed shorter after the initiation of symptoms (approximately 1 year) and was treated adding rituximab to glucocorticoids, obtaining excellent results and allowing us to spare glucocorticoid use, with no evidence of sequelae or residual symptoms.

IgG4-RD can be mimicked by other conditions, such as vasculitis, malignancies, other types of pancreatitis, Sjogren’s syndrome, sarcoidosis. Because of that, the diagnosis is often incorrect or delayed. To establish a more accurate diagnosis, characteristics of IgG4-RD mimics are collected as exclusion criteria in the 2019 American College of Rheumatology / European League Against Rheumatism classification criteria for IgG4-RD [15]. In our case, other autoimmune, infectious or malignant diseases were deeply investigated at the time of diagnosis and reasonably excluded.

## Conclusion

IgG4-RD is a rare inflammatory disease with a wide range of manifestations. Diagnosis is challenging, but evidence on clinical, serological, imaging and histological features is increasing. In addition, recent classification criteria have been developed to help standardize future research. We describe an uncommon presentation of IgG4-RD beginning in the larynx. We also highlight its excellent outcome after initiating adequate immunosuppressive therapy.
